# Highlights misleading the determination of prolonged fever etiology

**DOI:** 10.1186/1471-2334-13-S1-P103

**Published:** 2013-12-16

**Authors:** Natalia Cirlea, Daniela Desagă, Emilia Nicoară, Alexandru Crişan, Alexandra Sima, Flaviu Bob

**Affiliations:** 1 Department of Infectious Diseases II, “Victor Babeş” Clinical Hospital of Infectious Diseases Timişoara, Romania; 2 Department of Infectious Diseases, Municipal Emergency Hospital Timişoara, Romania; 3 “Victor Babeş” University of Medicine and Pharmacy, Timişoara, Romania; 4 Clinic of Diabetes, Nutrition and Metabolic Diseases, County Emergency Hospital Timişoara, Romania; 5 Clinic of Nephrology, County Emergency Hospital Timişoara, Romania

## Background

Of the myriad of disorders causing fever of unknown origin, infections have comprised the largest category (25-50% of cases) explaining frequent misinterpretation of some laboratory results.

## Case report

We present the case of a 34-year-old female with a medical history of fever lasting 1 week before admission to the County Hospital, Clinic of Diabetes, Nutrition and Metabolic Diseases for multiple-localized arthritis (bilateral tibia-tarsus, left radio-carpal, II and III metacarpus-phalanx right hand, right knee), considered reactive arthritis by the rheumatologist; these symptoms occurred a few days after a dental incision. The patient received 1 week antibiotic treatment for positive blood culture with *Staphylococcus aureus*, without any improvement. The synovial fluid culture revealed *Acinetobacter baumannii* and the patient was transferred to the Infectious Diseases Clinic II to continue the therapy. The patient followed a further 2-week treatment with a combination of antibiotics, according to the antibiotic test and remediation of dental foci, with decrease in fever and fluctuating evolution of joint inflammatory phenomena. The rheumatologist confirmed the opinion that it was a reactive arthritis. After the expansion of the antibiotic regimen and adding a nonsteroidal anti-inflammatory drug the patient developed acute renal failure that required a decrease in antibiotic dose and the transfer to the Nephrology Department. After the remission of renal failure, the patient underwent an MRI, because of the persistent fever and arthritis, which revealed sacroiliitis. Sacroiliitis proved to be the main cause of the prolonged fever and reactive arthritis.

## Conclusion

1. The persistence of fever, despite the appropriate antibiotic association treatment administered according to the antibiotic test for positive cultures, shows that the infection was not the etiology of the prolonged fever, and the presence of *Staphylococcus aureus* and *Acinetobacter baumannii* in the cultures was probably a contamination.

2. Sacroiliitis proved to be the main cause of the prolonged fever and reactive arthritis.

